# Bridging molecular insights and clinical application: non-coding RNAs, targeted drug delivery, and metastatic breast cancer therapy

**DOI:** 10.1007/s12672-025-03090-1

**Published:** 2025-07-26

**Authors:** Sohini Chakraborty, Satarupa Banerjee

**Affiliations:** 1 Department of Biotechnology, School of Applied Sciences, Reva University, Bengaluru, 560064 Karnataka India; 2 Department of Biotechnology, School of Biosciences and Technology, Vellore Institute of Technology, Vellore, 632014 Tamil Nadu India

**Keywords:** Breast cancer, Metastasis, Drugs, Drug resistance, Targeted drug delivery, Non-coding RNA

## Abstract

Breast cancer (BC) is one of the most common types of malignancy diagnosed globally. Metastasis plays a major role in most of the cancer-related mortality among affected patients. Despite the advances in the areas of early detection and localized treatment modalities, there prevail several challenges which the therapeutic strategies encounter, like drug resistance, tumor heterogeneity, and drug delivery. This review presents a comprehensive and detailed overview of organ-specific metastasis that occur in BC, specifically emphasizing key sites such as the bone, liver, lung, and brain. It also outlines the significance of various therapies like chemotherapies, endocrine therapies, targeted therapies and immunotherapies that have been clinically approved to date. The review specifically emphasizes the molecular mechanisms by which non-coding RNAs (ncRNAs) act to exert their effects in regulating drug resistance. It also addresses the new advances in nanotechnology-based drug delivery systems (DDS) that function to enhance the specificity of treatments while simultaneously reducing systemic toxicity. Beyond ncRNAs, this review also explores other critical mechanisms of drug resistance in metastatic BC, including efflux transporter activity, target gene mutations, and micro-environmental factors, to mention a few. Moreover, the review also discusses the clinical significance of combination therapies and new therapeutic strategies, including the use of repurposed drugs and the concepts of personalized medicine. A greater understanding of the ncRNA-mediated signaling pathways, in combination with the latest advances in drug delivery systems, has the potential to greatly improve therapeutic efficacy and could result in more favorable clinical outcomes in the treatment of metastatic BC (MBC).

## Introduction

Breast cancer (BC) is among the most prevalent and deadly types of malignancy among women globally. BC has emerged as the most prevalent malignancy, overtaking lung cancer, with new incidences reaching millions per year. Early detection methods, such as mammography and molecular characterization, have improved the outlook of patients with early-stage BC, whereas that of MBC remains abysmal. Metastasis—a spread of cancer cells from the primary tumor to distant sites—is responsible for most BC mortality [1]. According to GLOBOCAN statistics, in 2022, almost 500,000 deaths are attributed to BC worldwide. Contrasting data for 5 years reveal that in cases of regional lymph node metastasis, the overall survival (OS) rate for BC is 86%, while in the case of distant organ metastasis, the mortality rate is reduced to 29%. 65% of BCs are diagnosed at a localized stage with a respective 5-year OS rate of 99% [2]. MBC can be thought as a continuum of pathologies with heterogenous molecular subtypes, metastatic sites, and complex interactions with the tumor microenvironment. The most common sites of metastasis for BC include the bone, liver, lungs, and brain. All of these metastatic sites present unique therapeutic problems depending on the differences in vascularization, immune surveillance and drug penetration. Additionally, the ability of the tumor cells to adapt and acquire resistance to most therapeutic agents makes it a multivariate problem to be treated.

Successful treatment of MBC requires a clear understanding of the molecular basis of the disease and how tumor cells develop resistance to therapeutic drugs [3]. Resistance can be intrinsic, i.e., tumor cells are inherently resistant to certain treatments, or acquired, which is developed over time as a consequence of selective pressures caused by repeated drug exposure. Resistance mechanisms include drug target alterations, activation of backup signaling cascades, enhanced DNA repair mechanisms, immune escape, and the influence of ncRNAs, which have more recently come to be appreciated as critical regulators of gene expression and drug susceptibility [4]. In the review, a detailed account of existing and emerging treatments for MBC, with particular emphasis on resistance mechanisms is included. Various drug therapies tailored for specific metastasized locations such as the bone, liver, lung, and brain, drug delivery systems and outlines different pathways involving ncRNAs and signaling pathways that affect drug efficacy. In doing so, the review emphasizes on personalized medicine regimens and combinations towards drug resistance prevention. To make the review available to a broad scientific audience, we begin by establishing key concepts like the categorization and biological roles of non-coding RNAs, the mechanistic principles of cancer pharmacotherapies and the dominant mechanisms underlying the resistance development. Summarising all the features, this review bridges the gap between molecular insight and clinical translation, thus establishing a strategic roadmap for future research and therapeutic advancement in metastatic BC.

Various factors like reproductive (premature menarche, late menopause, nulliparity) [5, 6], genetic (risk of women having BC increases twofold with a family history of BC) [7], dietary and lifestyle (obesity, smoking, drinking) [8, 9], and environmental (carcinogens, industrial pollutants, long-term disrupted circadian rhythm due to work schedules, exposure to exogenous estrogen) [10] factors contribute to the predisposition and progression of BC. Due to treatment procedures like chemotherapy, radiotherapy, mastectomy, menopause, etc., [11–13] patients are more prone to depression, which in turn increases the risk of cancer relapse and BCM.

Chemotherapy employs agents that primarily target rapidly proliferating cells, inhibiting DNA replication and microtubule formation. Taxanes such as paclitaxel and DNA-damaging agents such as doxorubicin are some of the well-known chemotherapeutic drugs. Hormonal therapies in hormone receptor-positive BC work via receptor blockade or hormone suppression. Examples include tamoxifen and aromatase inhibitors. Targeted therapies (HER2 inhibitors, such as trastuzumab) interfere with specific drivers of cancer growth and inhibit the HER2 expression in cancer cells. Immunotherapies employ the immune system to target and destroy cancer cells more efficiently, with checkpoint inhibitors against PD-1 or PD-L1 [14, 15]. Nano-drug delivery system (DDS) comprising of nanoparticles, nano-capsules, liposomes, micelles, micro-emulsions, etc., is a budding research field due to their potential reduced toxicity and high drug bioavailability [16, 17]. Drugs can be dissolved, absorbed, covalently bound to the nanoparticle surface, encapsulated or embedded inside the nanocarriers. In addition to such advantages, they also enable drugs to target specific tumour sites by exploiting the high permeability and retention properties of tumors [18]. Combination of neoadjuvant chemotherapy, surgery, radiotherapy, adjuvant chemotherapy and/or endocrine therapy [19–22] help in treating and managing MBC.

In the human genome, proteins are encoded by less than 2% of DNA. Remaining constituent portions of the DNA is transcribed into ncRNAs that control gene expression. The ncRNAs are categorized based on size and function. MicroRNAs (miRNAs) are about 22 nucleotides in size with traits of repressing gene expression by degrading mRNAs or by inhibiting translation [23]. Long non-coding RNAs (lncRNAs) with more than 200 nucleotides, control gene expression by remodeling chromatin, controlling transcription, and post-transcriptional regulation [24]. Circular RNAs (circRNAs) are circular RNA loops that bind and repress miRNAs, indirectly controlling gene expression. In cancer, ncRNAs are often dysregulated, promoting tumorigenesis, metastasis, and drug resistance [25]. Certain miRNAs can repress tumor suppressors or activate oncogenes. Abnormal lncRNAs and circRNAs can regulate signaling pathways, promote epithelial-to-mesenchymal transition (EMT), and interfere with drug efficacy. It is important to know the functions of these ncRNAs in MBC to develop targeted strategies to reverse drug resistance. Therapeutic interventions can be compromised by resistance mechanisms such as altered pathways, drug target mutation, enhanced drug efflux, or reorganization of the tumor microenvironment. Knowledge of drug action and resistance pathways will be essential in exploring treatment efficacy in MBC patients.

This review aims to provide a comprehensive overview of current and future treatment strategies for MBC, with a focus on resistance mechanisms. We review therapies for different metastatic sites, DDSs and the influence of ncRNAs and other molecular resistance mechanisms on drug efficacy. The review also explores the potential of personalized medicine approaches and combination therapies to overcome drug resistance. This foundation brings molecular knowledge into clinical practice and directs future studies in metastatic BC. In contrast to conventional reviews on drug resistance or delivery systems independently, this review combines them. It deals with BC metastases' organotropism and targeted therapy, as well as combining progress in ncRNA, nanotechnology drug delivery and combination therapies. By combining mechanistic understanding and therapeutic implications, this review gives an integrated view of how molecular biology tools can be used to bypass drug resistance in MBC.

## Organ-tropism-specific therapeutic strategies

### BC Bone metastasis (BCBoM)

Treatment therapies for bone metastasis in the solid tumour include:

- Bone-targeting agents (BTA): zoledronic acid, pamidronate disodium, denosumab, and bis-phosphonates.
- Radiation therapy: prevents skeletal-related events (SREs) in patients with bone metastasis.
- Conventional therapies (Table 1).

**Table 1:** Conventional and targeted treatments for BCBoM

Type of treatment	Therapeutic options	Uses	Refs.
Conventional treatments for BC bone metastasis	Systemic endocrine therapy Systemic chemotherapy Systemic targeted therapy Adjuvant bone-targeted therapy Radiotherapy Surgical intervention Analgesics	disease control, skeletal-related events (SREs), metastasis prevention, bone loss, pain relief, and re-calcification, metastatic spinal cord compression control, and chronic pain relief	[28]
Targeted agents for BC bone metastasis	Arginine-glycine-aspartic acid (RGD) targeting ανβ3 integrin Bisphosphonates (BPs): alendronate and zoledronic acid	RGD peptide prevents metastasis by blocking osteoclast-mediated osteolysis Nano-particle-mediated drug delivery of the BPs prevents metastasis to the bones	

The spine is the most common site of bone metastasis other than ribs, skull, pelvis, femur, and humerus. Common ways to cure bone metastatic cancers involve medications to shrink, stop or slow down the growth of the tumour in the bone tumor microenvironment. About 45–75% of patients fall victim to functional disability every 3–4 months, 10–15% of patients have hypercalcemia, and 10–20% of patients suffer from long bone fractures [26]. Treatment options include symptomatic treatments to relieve pain and prevent fractures, along with improving functional disability. Interdisciplinary treatment is necessary for BCBoM management for pain management, orthopedics, and other medical specialities [27].

### Drug delivery systems (DDS)

**Fig. 1:**
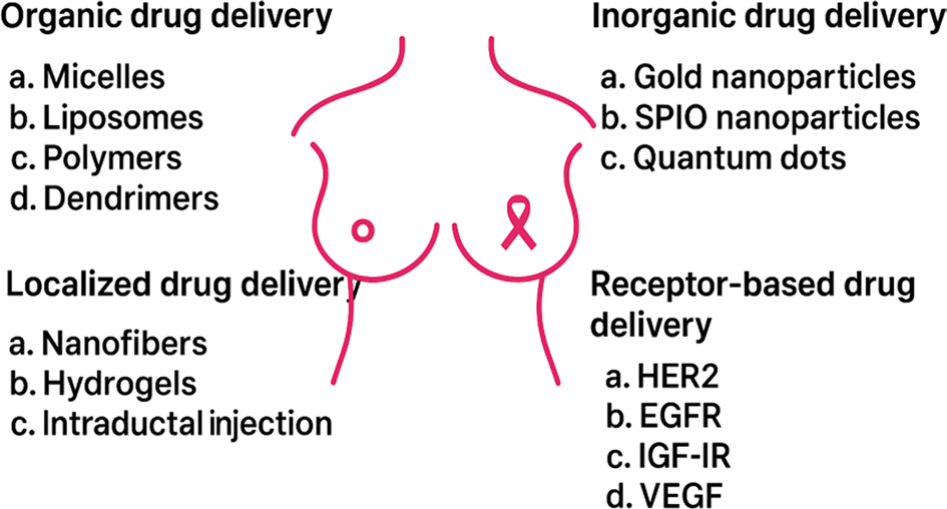
Different types of DDS in BCBM

Cisplatin poisoning can be avoided by combining the formula of the pH-sensitive properties and nanoparticles (DZ@ALN) released in an acidic bone microenvironment [29]. RGD-targeted dendrimer delivery of bortezomib to metastatic sites reduces the bone destruction of tibias to the most suppressive osteolysis in BC cells [30]. The types and media of DDS are illustrated in Fig. 1 and Table 2, respectively.

**Table 2:** Drug delivery media for BC bone metastasis

Loading compound	Target agent	Particle type	Uses	Refs.
Cisplatin prodrug	Alendronate	Polymeric nanoparticle (PN)	Relieves bone pain Prevent organs from cisplatin toxicity Prevent bone destruction Suppress osteolysis Reduced systemic toxicity Robust antitumor and anti-resorption activity	[9–27]
Cisplatin prodrug and Zoledronate				
Bortezomib and Curcumin				
Bortezomib and Zinc phthalocyanine				
Small molecule inhibitors of Gli2		Micelle		
Doxorubicin		Micelle, Nanosheet, Liposome and PN		
Bortezomib	Alendronate and RGD peptide	Micelle and Polymeric nanoparticle		
Docetaxel				
Paclitaxel		PN		
Gold nanorods	Zoledronic acid	Mesoporous silica nanoparticle		
Immunostimulatory oligonucleotide		Metal–organic framework nanoparticle		
Gadolinium		Mesoporous silica nanoparticle		
Iron oxide (Fe3O4) and indocyanine green		PN		

As studied in mice, the alendronate-modified bortezomib-catechol loaded prodrug micelles worked much better in an acidic environment. The ALN-NPs could significantly suppress tumour growth, resulting to bone decay [31]. Due to their increased bone affinity, curcumin-conjugated bortezomib-loaded NPs could suppress bone resorption and tumour growth. A high level of anti-bone resorption and increased bone volume was rendered by doxorubicin-loaded liposome [32]. Quinolone nonpeptide-conjugated ανβ3 integrin NPs were used to deliver docetaxel-prodrug to metastatic bone sites [33]. Paclitaxel-loaded pullulan delivery system could inhibit proliferation and metastasis due to its robust bone tissue affinity [34].

The primary symptom of BCBoM is back pain, including broken bones, and numbness caused by affected vertebrae pressurizing the spinal cord. Additionally, confusion, extreme thirst, loss of appetite, nausea and tiredness is caused by high levels of calcium in the blood [27]. The OS of patients with BCBM depends on ortho-bullets and the operative nature of the cancer. If operative, the OS is more than six months. When incurable, treatments include treating the original cancer, using drugs to prevent the breaking down of bones, strengthening weak bones by cement injection and relieving pain, and finally, going for surgery [35].

### BC liver metastasis (BCLiM)

Initially when BC cells migrate to liver, they do not show any symptoms. But with the help of the hepatic panel tests, doctors can pick up the signs and symptoms of liver metastasis. Among various signs and symptoms of liver metastasis, pain and discomfort in the mid-section is one of the primary along with fatigue and weakness. Weight loss due to poor appetite, fever and bloating, yellowing of skin with whites of eyes is also one of the primary symptoms. Therapies for BC liver metastasis is listed in Table 3.

**Table 3:** Therapeutic options and their efficacy against BCLiM

Treatment	Application and efficacy	Refs.
Local chemotherapy options	*Hepatic arterial infusion (HAI) chemotherapy* uses a small pump implanted under the skin in the lower abdomen to deliver delivers high doses of chemotherapy into the liver through the hepatic artery pump can be refilled with medication over time	[36]
	*Trans-arterial chemoembolization (TACE)* delivers micro beads filled with chemotherapy medication directly into the liver tumours micro beads travel through a small incision, guiding a tiny tube or catheter into the artery delivering the chemotherapy to the liver without damaging surrounding healthy tissues	
Hormonal therapy	Two ways of efficacy: a. lowering the amount of the hormone estrogen b. blocking the action of estrogen Medicines: Tamoxifen, Arimidex, Aromasin, Femara and Faslodex	
Targeted therapies	Less harmful than chemotherapy. Also called immune-targeted therapies	
Immuno therapy	Help our immune system to fight cancer cells more efficiently by: a. stopping or slowing down cancer cell growth b. stopping cancer cells from spreading c. killing cancer cells more efficiently	
Local treatments	*Surgery*: attempted if the cancer is HR + and liver is the only metastatic site, patient is in good health and there one or two of lesions that can be removed *Stereotactic body radiation therapy (SBRT):* targets high-dose beams of radiation at the cancerous areas in the liver *yttrium (Y-90) radioembolization*: delivers radiation therapy directly to the tumours in the liver *ablation and local chemotherapy*: a. cryoablation, which uses extreme cold b. RFA (radiofrequency ablation): uses high-frequency electrical currents c. NanoKnife: delivers pulses of electrical currents to the tumour and leads to cell death	

### BC lung metastasis (BCLM)

Available therapies for BC lung metastasis (BCLM) include cytotoxic chemotherapies, endocrine therapies and newly developed target therapies. Systemic chemotherapy has not proven to be of much advantageous to OS of patients [27]. BCLM symptoms may be similar to the flu or a cold. A persistent cough, lung pain, shortness of breath, wheezing, exhaustion, recurrent chest infections, coughing up blood, loss of appetite, and unintended weight loss are all signs of BCLM. BC cells must undergo major modifications to survive and proliferate in the lungs [37]. Additionally, these cells must adapt to combat immune system attacks. Depending on how the cancer metastasized, there will be different therapy choices. Systemic, or whole-body drugs that treat cancer all over the body are typically used as treatments for BCLM [35].

Some of them include:

- *Chemotherapy:* a pharmacological treatment that kills the body's rapidly dividing malignant and healthy cells [38].
- *Hormonal therapy:* a cancer treatment that slows the growth of cancerous cells by reducing the hormone levels the disease needs to thrive. BC with hormone receptors respond effectively to this therapy [39].
- *Targeted therapy:* cancer is allegedly treated more precisely than with chemotherapy. These therapies target particular proteins, chemicals, or receptors on cancer cells that either facilitate the body's immune system's ability to recognise and eliminate malignant cells or inhibit their growth [4].
- *Radiation:* to ease symptoms and slow the spread of metastatic BC, doctors frequently provide radiation therapy. Radiation therapy can aid in pain management and minimize the chance of broken bones in cancer patients [40].
- Various prescription drugs can help clean airways and lessen coughing. Others can help with pain, exhaustion, and loss of appetite. These include:
  Inhibitors of poly (ADP-ribose) polymerase (PARP) [41].
  Inhibitors of phosphoinositide-3 (PI-3) kinase [42].
  Circulating tumour cells and circulating tumour DNA after bevacizumab treatment (Avastin) [43].

Selumetinib, an MEK inhibitor is shown to inhibit lung metastasis of TNBC in xenograft models. Succinobucol (SCB) is a vascular cell adhesion molecule-1 (VCAM-1) inhibitor that suppresses BCLM too. A study by Sasha and colleagues utilized high throughput screening (HTS) of pro-metastatic drugs to understand various anti-metastatic drugs [44]. This work revealed that ponatinib is one such drug that can reverse express four BCLM-associated genes, namely, ANGPTL4, MMP1, PTGS2 and TNC*.* The underlying mechanism of ponatinib is by inhibiting c-Jun transcription factor (TF). Ponatinib represses the mRNA transcription and fuels c-Jun degradation via the ERK/c-Jun pathway.

### BC brain metastasis (BCBM)

In the case of BCBM, the HER2 expression statuses differ from those in primary tumours. This homogeneity of genetic alterations can be targeted and hence influences the treatment options in case of brain metastasis [45]. The blood–brain barrier (BBB) is another hindrance to BCBM treatment. Traditional therapies for BCBM include multiple model strategies like stereotactic radiosurgery (SRS), whole-brain radiotherapy (WBRT), palliative therapy and the most common chemotherapY [46]. BCBM patients suffer from poor quality of life (QoL) and insufficient prognosis. Novel therapies for BCBM include the reformulation of existing compounds alongside several immunotherapies and nanotherapies. Some of such therapeutic options are listed in Table 4. The five NPs currently existing for BCBM therapy include liposomes (60–500 nm), micelles (10–60 nm), dendrimers (5–250 nm), polymers (10–60 nm) and SPIO (iron oxide oleic acid amphiphilic polymers, 5–30 nm) [47].

**Table 4:** Efficacy for BCBM-targeted drugs

Drugs	Category	Application	Refs.
Trastuzumab, Pertuzumab	Monoclonal antibodies	Anti-HER2 drugs	[48]
T-DM1		Antibody–drug conjugate	[46]
Cetuximab, Panitumumab		EGFR-targeted drugs and molecules	[49]
Erlotinib, Gefitinib, Afatinib		Tyrosine kinase inhibitors	
lapatinib, neratinib, tucatinib	Small molecules		[50]
Docetaxel	Oral drug	Anti-mitotic drug	[51]
Tucatinib		HER2-specific tyrosine kinase inhibitor	[52]
Pyrotinib		Irreversible pan-ERBB inhibitor targeting HER1, HER2 and HER4	[53]
Olaparib, Rucaparib, Niraparib, Veliparib and Talazoparib		PARP inhibitors	[54–56]
Ramucirumab		Human immunoglobulin G1 antibody	[57]
Biparatopic anti-HER2 (bHER2-ATC)	Antibody–tubulysin conjugate	Inhibited tumour growth in a murine BCBM model by brain penetration	[58]
GDC-0084	Dual PI3K–mTOR inhibitor	Penetrate the BBB	[59]
GDC-0068	pan-Akt inhibitor		[60]
Everolimus	Rapamycin (mTOR) inhibitor		[56]

**Fig. 2:**
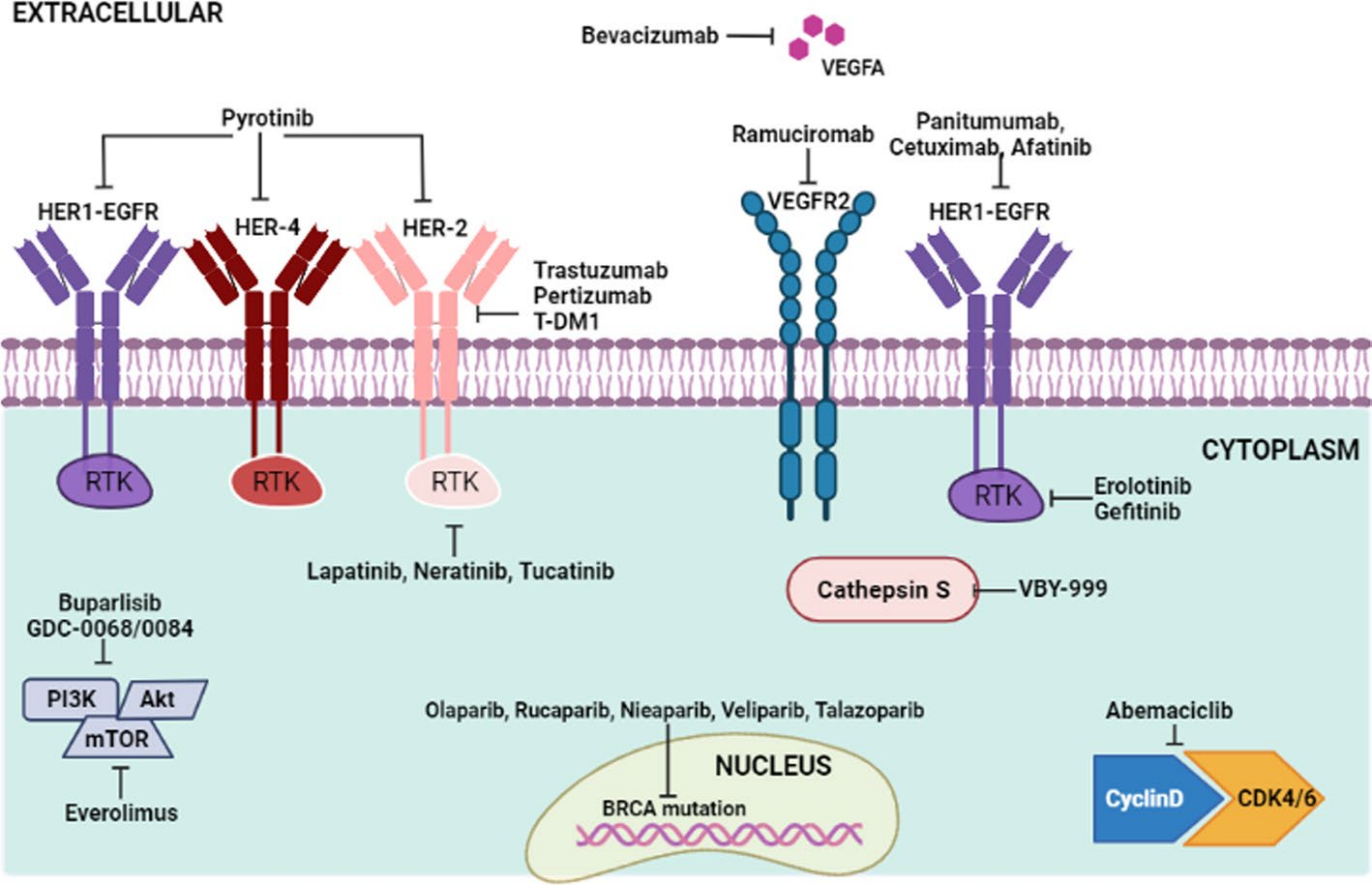
Novel targeted therapeutics in BC brain metastasis

Table 5 elaborates some of the drugs which are highly rated drugs for BCM treatment. Figure 2 schematically represents the mechanism of action of different drugs involved in BCBM. Trastuzumab emtansine (T-DM1) is more effective than capecitabine combined with Lapatinib. Lapatinib inhibits efflux transporters on BBB [50]. When combined with chemotherapeutic agents, Lapatinib has increased efficacy against brain metastasis [61, 62]. Afatinib and Capecitabine are referred to as the first-line of treatment for untreated HER2 + BCBM. Neratinib combined with capecitabine has higher therapeutic effect against refractory HER2 + BCBM [63]. When combined with Trastuzumab and Capecitabine [52], Neratinib works against nausea, diarrhoea, fatigue and overexpressed aminotransferase. Everolimus in combination with Buparlisib (PI3K inhibitor) clinically works against brain metastasis. Also, the combinatorial treatment of everolimus, lapatinib and capecitabine in HER2 + BC brain metastatic patients [56] exhibits anti-tumour effect.

**Table 5:** Metastatic drugs in BC with ratings more than ‘6’

Drug	Utility	Warning	Prerequisite	Drug administration	Dosage	Side effects	Refs.
Ibrance	Interferes with the growth and spread of HER2 cancer	It might affect the lungs/immune system	*Women*: not to be used if pregnant Birth control is to be used for at least 3 weeks post-last dosage *Men*: birth control for at least 3 months post-last dosage	Generally, a 28-day treatment cycle Swallow whole capsules without crushing (with/without food), avoid grape-food products To be taken at the same time every day	125 mg orally per day for 21 consecutive days followed by 7 days off	Blisters/ulcers in the mouth, low blood counts, inflammation and blood clotting in lungs, fatigue, dry skin/rash, altered taste	[64]
Letrozole	Lowers estrogen levels in postmenopausal women, often given to women who have been taking tamoxifen for 5 years	Not to be taken if pregnant, can cause harm to the unborn baby	Birth control to be used for at least 3 weeks post last dosage	With/without food, frequently checked bone mineral density Room temperature storage, no contact of moisture and heat	25 mg orally/day *Adjuvant* to be discontinued at relapse, *Advanced* until tumour progression	Redness in face or chest, headache, bone/muscle pain, weight gain, increased cholesterol in the blood	
Femara	Lowers estrogen levels in postmenopausal women, often given to women who have been taking tamoxifen for 5 years	Cannot be taken if a patient has osteoporosis, high cholesterol, or liver disease Since the drug impairs thinking, activities that require being alert need to be avoided	Birth control if you are not past menopause. Keep using birth control for at least 3 weeks after your last dose of Femara	Once/day, with/without food, frequently checked bone mineral density Room temperature storage, no contact with moisture and heat	*Anti-estrogen* 2.5 mg tablet orally/day *advanced* Letrozole therapy should continue until tumour progression On relapse, it should be discontinued General Adult dose: adjuvant	Redness in face or chest, headache, bone/muscle pain, weight gain, increased cholesterol in the blood	
Anastrozole	Lowers estrogen levels in postmenopausal women, often given to women who have been taking tamoxifen for 5 years	Decreases blood flow in the heart, history of coronary artery disease, medical attention needed in case of new chest pain or shortness of breath	approved for use in men or children not to be used allergic to it, or have not yet completed menopause History of heart problems, high cholesterol, osteoporosis	Once/day, with/without food, need to take the medication for up to 5 years Room temperature storage, no contact with moisture and heat	Initial dose: 1 mg orally taken once/day until tumour progression	Allergic reactions, numbness in wrists, bone fracture symptoms, signs of stroke, depression and sleep problems, rash, nausea	
Faslodex	An anti-estrogen drug used alone or in combination with Ibrance and Verzenio	Avoided if the history of liver disease, bleeding problems, and thrombocytopenia Not to be used in men	Should have a negative pregnancy test at least 7 days before treatment	Two injections into a muscle of your buttock can take up to 2 min to complete/injection usually given once every 2 weeks at first, and then once a month	Monotherapy: 500 mg IM into the buttocks (gluteal area) slowly (1 to 2 min per injection) as two 5 mL injections, one in each buttock, on days 1, 15, 29 and once monthly thereafter Combination Therapy: In combination with palbociclib or abemaciclib: 500 mg IM into the buttocks (gluteal area) slowly (1 to 2 min per injection) as two 5 mL injections, one in each buttock, on days 1, 15, 29 and once monthly thereafter	Affects fertility in both men and women, pain at the administration site, arms, legs, bone, joints, constipation, abnormal liver function tests	
Arimidex	Lowers estrogen levels in postmenopausal women	Decreases blood flow in the heart, history of coronary artery disease, If taken during pregnancy, it might harm the unborn child It might not work well with other estrogen medication Might increase the risk of blood clotting or stroke	Not approved to be used in men and children Not to be taken with tamoxifen Avoid if there is a history of coronary artery disease, high cholesterol, or osteoporosis Might weaken bones	Once/day. With/without food Might be necessary for almost 5 years Room temperature storage, no contact with moisture and heat	Initial dose: 1 mg orally once/day until tumour progression detected	Avoid in case of allergic reactions or severe skin reactions Shortness of breath, numbness in wrists, liver problems, symptoms of stroke	
Herceptin (trastuzumab)	Commonly used to treat metastatic BC	Not in pregnancy Avoid pregnancy for at least 7 months post the last dosage Can cause heart failure	Not to be used in patients with a history of heart diseases, congestive heart failure, heart attack or allergies	Intra-venous Once/week or once/1–3 weeks Heart functions need to be checked both before and after treatment Also, every 6 months heart functionality needs to be monitored for 2 years after the last dose Usually given for 52 weeks	Used alone or in combination with paclitaxel Initial dose: 4 mg/kg IV infusion over 90 min Subsequent therapy: 2 mg/kg IV infusion over 30 min once weekly until disease progression	It might occur during injection It might cause worsening cough, blurred vision with headache, blisters in the mouth, heart anomalies, low blood cell counts, tumour breakdown symptoms	
Palbociclib	Treats HER2- BC in both men and women In postmenopausal women, palbociclib is given in combination with a hormonal medicine Otherwise, palbociclib is given in combination with fulvestrant	It might affect the lungs or immune system	Avoided if there is a history of liver or kidney disease Women should use birth control for at least 3 weeks after post last dose Men should use birth control for at least 3 months after post last dose	28-day treatment cycle need to take the medicine during the first 3 weeks of each cycle Take uncrushed/unbroken capsules with food. Avoid grapefruit products Room temperature storage, no contact with moisture and heat	125 mg orally/day for 21 consecutive days followed by 7 days off	Blisters or ulcers in the mouth, low blood cell counts, Signs of inflammation in the lungs, and blood clotting symptoms in the lungs	
Taxotere	Disrupts growth and spread of BC Lowered blood cells help the body to fight infections and help blood clotting	Life-threatening allergies, and swelling in the intestines eventually cause death Steroids to be used to avoid fluid retention	This should be avoided if the patient has a low WBC count or a history of a severe allergic reaction History of liver and heart disease, fluid retention, tumour lysis syndrome, alcohol consumption *Women*: birth control at least for 6 months post-last dosage *Men*: birth control at least for 3 months post-last dosage	Infused into the vein Medical reporting for any burning sensation, swelling around the IV needle	locally advanced/ metastatic post the failure of prior chemotherapy: 60 mg/m^2^ to 100 mg/m^2^ iv over 1 h every 3 weeks Adjuvant treatment: 75 mg/m^2^ 1 h after 50 mg/m^2^ doxorubicin & 500 mg/m^2^ cyclophosphamide every 3 weeks for 6 courses. Prophylactic G-CSF: to avoid the risk of haematological toxicities	Signs of tumour cell breakdown, the feeling of being drunk, liver problems and low blood cell counts, muscle weakness in arms, legs, feet or hands, swelling and numbness in hands and feet or face, constipation, diarrhoea, eye redness	
Halaven	Treat BCM Also, liposarcoma	Not in pregnancy Suppress the immune system Might get an infection or bleed Avoid being near people who are sick or with infections	Report any history of liver/ kidney/ heart disease, long QT syndrome, electrolyte imbalance Women: birth control at least for 2 weeks post last dose Men: birth control at least post 3.5 months post last dose	21-day treatment cycle Medication to be taken only during 1–2 weeks of each cycle Heart infection may also need to be checked using an electrocardiograph	1.4 mg/m2 IV over 2 to 5 min on days 1 and 8 of a 21-day cycle	Low calcium, and potassium level Low blood cell counts Burning sensations during urination, nausea, hair loss, feeling tired or weak	
Gemzar	Treat BCM Also, to treat cancers of the pancreas, lung, ovary, and breast	Might lower blood cells; helping the body to fight infections Might also affect the lungs, kidneys or lungs Might have stomach pain, little or no urination, dark urine, yellowing of eyes or skin,	History of kidney/ liver disease, alcoholism, radiation treatment Women: birth control for at least 6 months post-last dose Men: birth control for at least 3 months post last dose	Intravenous, report any kind of swelling or burning pain around the iv Changes immune system result in an increased risk of bleeding or infection		Liver problems, low blood cell counts, fluid build-up in or around the lungs, damaged RBCs, fever, mild rash, swelling in hands or feet, shortness of breath, reddish or pinkish urine	
Trastuzumab	Treat metastatic BC Also, treats metastatic stomach cancer	Might cause heart failure Prior reporting of any existing heart disease,	History of heart disease/attack/failure Any allergies or breathing problems Not to be pregnant Has to be under birth control for at least 7 months post the last dose	Intra-venous once every week or every 1 to 3 weeks medicine must be given slowly, and the infusion can take up to 90 min to complete	52 weeks. heart function may need to be checked before and during treatment need heart function testing every 6 months for 2 years after your last dose	Signs of an allergic reaction: hives, difficulty breathing, swelling of your face, lips, tongue, or throat. New worsening cough, heart problems, low blood cell counts, signs of tumour cell breakdown	
Eribulin	Treat metastatic BC and liposarcoma	suppress your immune system and you may get an infection or bleed more easily	Liver/kidney disease, heart problems, long QT syndrome, electrolyte imbalance Woman: birth control for at least 2 weeks post last dose Men: birth control for at least 14 weeks post-last dose No breastfeeding while on medication and at least 2 weeks post last dose	Intra-venous	21-day treatment cycle use the medicine only during the first 1 to 2 weeks of each cycle to suppress the immune system, may get an infection or bleed more easily blood tested for heart function to be checked using an electrocardiograph	Low calcium, and potassium levels Low blood cell counts Burning sensations during urination, nausea, hair loss, feeling tired or weak	
Paclitaxel	Treat metastatic BC, ovarian cancer, lung cancer and AIDS-related Kaposi’s sarcoma	signs of an allergic reaction: hives; difficulty breathing; feeling like you might pass out; swelling of your face, lips, tongue, or throat lower blood cells that help your body fight infections	Check WBC counts, report any known allergy to castor oil, heart/liver problems Not in pregnancy	Intravenous, 175 mg/m2 IV over 3 h every 3 weeks given slowly, and the infusion can take 3 to 24 h to complete once every 2 to 3 weeks	Severe stomach pain/diarrhoea, cold symptoms, flushing, pain during urination, severe headache, low WBCs, and RBCs		
Ribiciclib	Treats HER2-BC Generally given in combination with letrozole or fulvestrant	chest pain, fast or pounding heartbeats, trouble breathing, cough (with or without mucus), sudden dizziness, right-sided upper stomach pain, loss of appetite, unusual bleeding or bruising, dark urine, or yellowing of your skin or eyes	Allergic reactions, slow heartbeats, heart disease or prior heart attack, long QT syndrome, electrolyte imbalance, liver/ kidney disease,	A 28-day treatment cycle. You will take the medicine for the first 21 days of each cycle, followed by 7 days off Swallow the tablet whole, and do not crush, chew, or break it simultaneously each morning, with or without food	Fast or pounding heartbeats, fluttering in your chest Low white blood cell counts, signs of lung inflammation and liver problems. Cough, nausea, diarrhoea, headache, rash, hair loss		
Gemcitabine	Treats BC, apart from pancreatic cancers, cancers of the lungs and ovary	Increased bleeding or infection risk also affects your liver, kidneys, or lungs can increase your risk of bleeding or infection	Report kidney/liver disease. Alcoholism, radiation therapy Women: birth control for at least 6 months after your last dose Men: birth control for at least 3 months after your last dose	Intra-venous Might feel any burning, pain, or swelling around the IV needle	Allergic reactions, blisters in the mouth, severe skin redness, liver problems, low blood cell counts, fluid build-up, anxiety, damaged RBCs	stomach pain, dark urine, yellow skin or eyes, little or no urinating, swelling, rapid weight gain, severe shortness of breath, wheezing, or cough with foamy mucus	
Kisqali, Lynparza Bevacizumab Olaparib	Treat HER2- BC	serious side effects on your heart, liver, or lungs chest pain, breathing, cough, sudden dizziness, loss of appetite, dark urine, or yellowing of your skin or eyes	perform blood tests heart disease or prior heart attack, long QT syndrome, electrolyte imbalance liver disease, kidney disease, not to be used in pregnancy	28-day treatment cycle. medication for the first 21 days of each cycle, followed by 7 days off Swallow the tablet whole and do not crush, chew, or break it In the morning, with or without food	600 mg orally once a day	May affect fertility in both men and women, Allergies, severe skin reaction, low white blood cell counts, signs of inflammation in the lungs, liver problems, nausea, constipation, rash, hair loss

### BC subtype-specific drugs and therapies

Despite promising advances in the laboratory and preclinical settings, the clinical translation of novel therapeutics in MBC faces several hurdles that limit their real-world impact. While numerous clinical trials have evaluated targeted agents, immunotherapies, and ncRNA-based strategies, only a fraction successfully progresses to regulatory approval or widespread clinical use [65].

One major challenge lies in the heterogeneity of metastatic tumors [66], which exhibit diverse genetic and epigenetic profiles not only between patients but also within different metastatic sites of the same individual. This complicates the identification of universally effective therapies and complicates patient stratification in clinical trials. Moreover, many trail limitations have relatively small sample sizes and lack long-term follow-up, reducing the generalizability and robustness of their conclusions.

In the case of targeted therapies and immunotherapies, while some agents have shown remarkable efficacy in specific subgroups—such as HER2-positive or PD-L1-expressing tumors—other trials have failed to demonstrate benefit in unselected patient populations. Targeted therapies for example, PI3K inhibitors like alpelisib have been approved for use in PIK3CA-mutant hormone receptor-positive MBC, but they are associated with toxicity and modest progression-free survival benefits [67]. Similarly, immune checkpoint inhibitors such as atezolizumab have shown benefit only in PD-L1-positive triple-negative breast cancer, with limited durability and inconsistent outcomes across trials [68].

Another limitation is the delivery and stability of novel therapeutics, particularly those based on RNA molecules. ncRNA-based therapies, including miRNA mimics and lncRNA inhibitors, face significant barriers related to degradation by nucleases, inefficient cellular uptake, and off-target effects. Although delivery systems such as lipid nanoparticles and exosome-based platforms are under investigation, few have successfully reached late-phase clinical trials [69].

Additionally, the development of resistance to these new therapies often mirrors the same issues seen with traditional drugs. Cancer cells rapidly adapt to targeted inhibition by activating compensatory pathways, altering the tumor microenvironment, or evolving under immune pressure. This underscores the need for combination therapies and longitudinal monitoring of molecular markers during treatment. To enhance clinical translation, it is critical to adopt adaptive trial designs, expand biomarker-driven studies, and utilize real-time genomic profiling. Moreover, the inclusion of patient-derived xenografts and organoid models in preclinical testing can improve the predictive power of translational research. Ultimately, interdisciplinary collaboration between bench scientists, clinicians, pharmacologists, and bioinformaticians is essential to bridge the gap between experimental promise and clinical reality in the treatment of MBC. BC subtype can help detect high risk for certain metastatic sites that can be tailored for personalized medicine and aim at better therapeutic strategies (Table 6).

**Table 6:** Clinically approved drugs for BC subtypes

Drug (commercial name)	Subtype/stage	Mechanism of action	Common side effects
CDK4/6 inhibitors	ER+, PR+, HER2-, Advanced/Metastatic	Inhibits cell cycle progression	Neutropenia, nausea, anemia, fatigue, diarrhea, vomiting
Abemaciclib (Verzenio)			
Palbociclib (Ibrance)			
HER2-targeted therapies	HER2+, early/advanced	Inhibits HER2 signaling	Diarrhea, liver problems, nausea, fatigue, cardiotoxicity
Trastuzumab (Herceptin)			
Pertuzumab (Perjeta)			
Ado-trastuzumab emtansine (Kadcyla)			
Neratinib (Nerlynx)			
Lapatinib (Tykerb)			
Hormonal therapies	ER+, PR+, variable stages	Lowers estrogen activity or blocks ER	Hot flashes, mood swings, bone loss, blood clots
Anastrozole (Arimidex)			
Exemestane (Aromasin)			
Fulvestrant (Faslodex)			
Raloxifene (Evista)			
Tamoxifen (Nolvadex)			
Chemotherapy (Taxanes and Anthracyclines)	Early/advanced	Disrupts mitosis or damages DNA	Hair loss, nausea, leukopenia, neuropathy, irregular periods
Paclitaxel (Taxol)			
Docetaxel (Taxotere)			
Doxorubicin (Adriamycin)			
Epirubicin (Ellence)			
Daunorubicin (Cerubidine)			
DNA damaging agents	Advanced	DNA alkylation or damage	Fatigue, nausea, neuropathy, kidney issues
Carboplatin (Paraplatin)			
Cyclophosphamide (Cytoxan)			
Vincristine (Oncovin)			
Antimetabolites	Metastatic/advanced	False nucleotide incorporation	Diarrhea, neutropenia, hand-foot syndrome
Capecitabine (Xeloda)			
Gemcitabine (Gemzar)			
Fluorouracil (Adrucil)			
mTOR/PI3K pathway inhibitor	HER2+, HER2-	Inhibits mTOR kinase	Fatigue, mouth sores, infections, diarrhea
Everolimus (Afinitor)			
Immunotherapy	TNBC, PD-L1+	Boosts immune response	Immune-related toxicities, fatigue, diarrhea
Atezolizumab (Tecentriq)			
Supportive agents	Various/combo	Prevent bone loss, stimulate blood cells	Bone pain, fever, GI symptoms, injection site reactions
Denosumab (Xgeva)			
Zoledronic acid (Zometa)			
Filgrastim (Neupogen)			
Pegfilgrastim (Neulasta)			
Epoetin alfa (Epogen/Procrit)			
Darbepoetin alfa (Aranesp)			

### Non-coding RNAs mediated drug resistance in metastatic BC

**Fig. 3:**
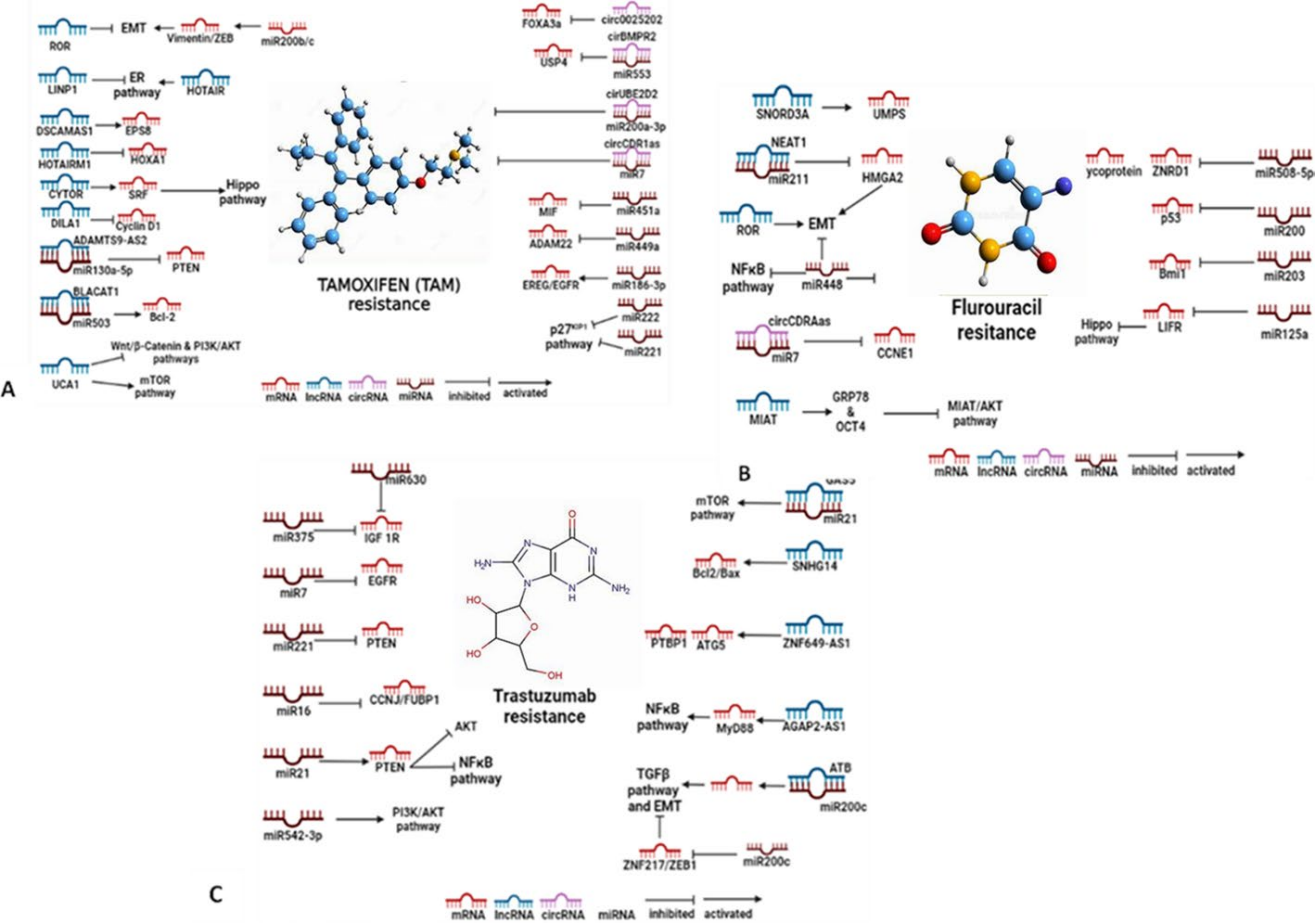
Classes of ncRNAs rendering resistance against. **a** Tamoxifen. **b** Fluorouracil and **c** Trastuzumab

The ncRNAs can modulate cancer cell therapeutics. Some drug classes like anthracyclines, taxanes, docetaxel, cisplatin and fluorouracil are commonly used to treat BC. Increased resistance against these drugs owed to the different classes of ncRNAs, which results in tumour progression and resistance to the therapies. Below (Tables 7, 8, and 9; Fig. 3a–c) listed are some of the underlying mechanisms of how ncRNAs mediate drug resistance.

**Table 7:** ncRNAs in anthracycline resistance

mi/lnc-RNAs	Target drugs	Property	Mechanism	Refs.
miR200c	Doxorubicin in MCF-7 human cell lines	Epirubicin Chemoselectivity	By inhibiting p-glycoprotein	[10, 11]
miR34a miR181b	Adriamycin (ADM) in MCF-7/ADR cells Tumour development	Sensitivity Oncogenic and chemoresistance	NOTCH1 pathway arrest	[12, 13]
miR302S family (a/b/c/d)	Adriamycin in MCF-7/ADR cells	Increased sensitivity of BC cells for ADM	Activation P-glycoprotein MAPK/ERK	[15]
miR148/152 family miR124-3p	Adriamycin	Resistance Sensitivity	Inhibition Spindlin1 and increases of UGT2B4, CYP2C8, UGT2B17, and ABCB4 Inhibition ABCC4	[16] [17]
miR29a		Resistance	PTEN/AKT/GSK3β inhibition	[19]
miR130b		Resistance, promotes proliferation	PTEN and PI3K/AKT targeting	[20]
miR222		Decreased sensitivity; ADM Resistance	Inhibition PTEN/AKT/p27^KIP1^ pathway	[21]
miR489	Adriamycin in MCF-7/ADM cells	Sensitivity	Inhibition EMT/Smad3	[23]
miR-221	Adriamycin	Sensitivity; can be used as a biomarker	Inhibition to HR	[26]
lncRNA-00518		Resistance	Inhibition miR-199a/ multidrug resistance protein 1 (MRP1) axis	[27]
lncRNA-GAS5			Suppressed Wnt/β-Catenin via miR-221-3p/DKK2 axis	[31]
miR298	Doxorubicin		Inhibition of P-glycoprotein	[18]
miR-760			Inhibition EMT/Nanog	[24]
miR-145		Sensitivity	Inhibition of MRP1 by targeting its 3’-UTR	[22]
miR-192-5p			JNK/Bad/Caspase9 activation Bcl-2/PPIA inhibition	[25]
lncRNA-LINP1		Resistance to 5-fluorouracil	decreased p53 effects	[28]
lncRNA-BORG		Resistance in TNBC cell lines	NF-κB pathway activation	[30]
LncRNA-NONHSAT101069	Epirubicin	Resistance	Acts as ceRNA with miR-129-5p/Twist1 targeting/EMT induced metastasis	[29]

**Table 8:** ncRNAs in paclitaxel resistance

mi/lnc/circ-RNAs	Target drugs	Property	Mechanism	Refs.
mi125b	Paclitaxel	Resistance	Bcl2 antagonist killer 1 (BAK1) inhibited, miR-125b, 221,223 and 923 upregulated	[61]
mi520h			death-associated protein kinase 2 (DAPK2) inhibited	[62]
mi451			Bcl-2 inhibited	[63]
mi100 mi18a		Sensitivity Resistance	mTOR pathway inhibition	[65] [66]
mi101		Sensitivity in TNBC	myeloid cell leukemia-1 (MCL-1) inhibition	[67]
lin28		Resistance, Stemness to BC stem cells	Rb, p21 activation let7 inhibition by targeting caspase 3	[57, 58]
let7a		Resistance IN MCF-7 BC cell lines	Caspase3 is inhibited by targeting thrombospondin -1(TSP1)	[59]
lncRNA-CASC2		Resistance	miR-18a-5p/CDK19 inhibition	[70]
lncRNA-H19		Resistance for Erα + BC, TNBC	AKT/BIK inhibition	[72–74]
lncRNA-00839		Resistance by binding to lin28B	PI3K/AKT pathway activation	[75]
CircRNA-ABCB10		Resistance by binding to let7a-5p	let-7a-5p/DUSP7 axis inhibited	[77]
circRNA-RNF111		Resistance	miR-140-5p/E2F3 inhibited by sponging of miR140-5p	[78]

**Table 9:** ncRNAs in docetaxel resistance

mi/lnc/RNAs	Target drugs	Property	Mechanism	Refs.
miR-141	Docetaxel	Sensitivity	EIF4E/CP110 activated	[82]
lncRNA-EPB41L4A-AS2			ABCB1 activation	[92]
miR-125a-3p			BRCA1 inhibited	[90]
miR-129-3p		Resistance	CP110 inhibited	[83]
miR-3646			GSK-3β/β-catenin pathway activated	[84]
miR-452			APC4 inhibited	[85]
miR-663			HSPG2 inhibited	[86]
miR-139-5p			Notch1 inhibition	[88]
miR-222/29a			Akt/mTOR activated	[20]
lncRNA-NEAT1			Sox2/ALDH activated	[93]

## Other mechanisms involved in drug resistance in metastatic BC (MBC)

Though drug resistance modulation in MBC has ncRNAs at the core, various other biological processes are important for the failure of therapeutic response. These resistance pathways tend to work together and support the heterogeneous and adaptive nature of BC growth. The following sections detail the important categories of resistance mechanisms deserving mention in an in-depth review of drug resistance in MBC.

### Efflux transporter-mediated resistance

A prominent mechanism contributing to multidrug resistance in MBC is the heightened expression of ATP-binding cassette (ABC) transporters [70]. These membrane-associated proteins function by actively extruding chemotherapeutic agents from cancer cells, thereby diminishing the intracellular concentration of the drugs and their therapeutic effectiveness. Significant transporters comprise:

- P-glycoprotein (ABCB1) [71] is responsible for resistance to anthracyclines, taxanes, and vinca alkaloids.
- MRP1 (ABCC1) [71] confers resistance to doxorubicin and etoposide.
- BCRP (ABCG2) [71] mediates resistance to mitoxantrone and topotecan.

### Target gene mutations and alterations

Drug resistance may be the result of genetic alterations in the drug target, resulting in decreased drug binding or altered downstream signaling.

ESR1 mutations are also present in endocrine-resistant ER + MBC and lead to ligand-independent receptor activation of the oestrogen receptor [72]. HER2 truncations (e.g., p95HER2) cause resistance to trastuzumab through loss of the extracellular binding domain. PIK3CA mutations are common in hormone receptor-positive BC and are responsible for resistance to PI3K/AKT/mTOR inhibitors [53].

### Enhanced DNA repair mechanisms

Resistance to DNA-damaging treatments like platinum-based chemotherapy and PARP inhibitors typically arises due to overactivated DNA repair mechanisms [73]. Homologous recombination repair (HRR): Reversion mutations in BRCA1/2 restore the competency of HRR, negating the action of PARP inhibitors. Mismatch repair (MMR): Overactivation may be the cause of resistance in certain subtypes [74].

### Tumour microenvironment (TME) influences

The tumour microenvironment impacts drug response dramatically by establishing drug-protective niches. Hypoxia enhances survival signals and suppresses reactive oxygen species produced by some chemotherapies [75]. Growth factors and cytokines released by cancer-associated fibroblasts (CAFs) shield tumour cells. PD-L1 modulation or immune cell recruitment of immunosuppressive cells (e.g., Tregs, MDSCs) decreases the efficacy of immunotherapy [67].

### Epithelial-to-mesenchymal transition (EMT) and cancer stem cells (CSCs)

EMT cells develop invasive potential and increased drug resistance. CSCs [76], often of EMT origin, are inherently resistant to traditional treatments due to low rate of proliferation, increased DNA repair activity and overexpression of drug efflux pumps.

### Metabolic reprogramming

Cancer cells repurpose their metabolism to grow in therapeutic stress conditions in general. Resistance can arise from augmented glycolysis (Warburg effect), dysregulation of mitochondrial function, lipid metabolism changes, most prominently in triple-negative BC (TNBC).

Clinical Implications and Strategies for Breaking Resistance Explanation of such resistance mechanisms offers the promise of therapeutic intervention. Interventions include combination therapy (e.g., PI3K inhibitors plus endocrine therapy for ESR1-mutant cancers) [67], next-generation inhibitors (e.g., tucatinib, which inhibits HER2 with improved BBB penetration), efflux pump inhibitors as chemotherapy adjuncts, and immunomodulatory drugs that target the reprogramming of the tumor immune microenvironment [77].

miR-21 also targets the tumor suppressor gene PTEN, which activates the PI3K/Akt cell survival pathway, leading to drug resistance to drugs such as trastuzumab and tamoxifen [78]. Similarly, miR-125b targets pro-apoptotic genes such as BAK1 and, by inhibiting chemotherapy-induced apoptosis, leads to resistance to drugs such as paclitaxel [79]. Loss of tumor-suppressive microRNAs such as miR-34a leads to overexpression of anti-apoptotic proteins such as BCL-2, which further leads to resistance [80]. lncRNA HOTAIR epigenetically silences tumor suppressor genes by recruiting polycomb repressive complex 2 (PRC2), augmenting doxorubicin resistance [81]. lncRNA UCA1 sequesters miR-143 [82], resulting in enhanced expression of multidrug resistance-associated protein and resistance to tamoxifen. GAS5 is, however, a tumor-suppressive lncRNA that sensitizes cells to chemotherapy by counteracting the miR-222/PI3K-Akt pathway [83]. circ-UBAP2 is another circRNA that sponges miR-661, resulting in the upregulation of ABC transporters, which increase the efflux of chemotherapeutic drugs like adriamycin.

These ncRNAs control numerous drug resistance-related pathways, such as PI3K/Akt/mTOR, Wnt/β-catenin, NF-κB, MAPK/ERK, and TGF-β. ncRNAs also impact the epithelial-to-mesenchymal transition (EMT), which is associated with metastasis and chemoresistance. ncRNAs also control the DNA damage response through genes involved in homologous recombination and base excision repair, thus impacting the sensitivity to DNA-targeting agents such as PARP inhibitors.

By regulating these molecular circuits, ncRNAs adjust the cellular response to treatment and enable adaptation to therapeutic stress. Dysregulation allows tumor cells to resist apoptosis, survive during drug treatment, and preserve proliferative potential. Therefore, inhibition of oncogenic ncRNAs or restoration of the activity of tumor-suppressive ones is a promising approach to overcoming drug resistance and improving treatment efficacy in metastatic breast cancer.

#### Aiduqing (ADQ) formula

In a study by Yang and colleagues, the anti-oncogenic role of ADQ formula is explored. Western blot results showed that treating high MBC cell lines like MDA-MB-231 and BT-549 with ADQ formula downregulated LC3-II, Beclin-1 and p-AMPK expression levels while p62 and p-mTOR were upregulated. Treatment of BC metastatic cells with ADQ formula not only deregulated starvation-induced metastasis but also derails autophagy by targeting CXCL1 and the invasion of metastatic cells into the tissues of nearby or distant organs, thereby inducing apoptosis of high MBC cells. These effects of the ADQ formula on BC metastatic cells reveal that it is a potential natural anti-metastasis medicine to be processed for clinical development and use [84–86].

#### Wenshen Zhuanggu (WSZG) formula

It is a complementary and alternative herbal drug comprising of coumarins such as osthole, xanthotoxin, bergapten used in adjuvant therapies for MBC, owing to the anti-cancerous effects of coumarins. Previous studies explored the role of signaling crosstalk of Jagged1/NOTCH-TGFβ-IL6 pathways in BC bone metastasis. This study by Chen and colleagues, reveal that WSZG reduces BCBoM and proliferation by targeting the Jagged1/NOTCH-TGFβ-IL6 axis [87, 88].

## Drug combinations approved for BC therapies

Combination therapies, also known as polychemotherapy, have been used for a long time now. Polychemotherapy has proven to be much more effective than single-agent treatment [89]. Traditional combination treatment for BC involves a combination of an alkylating agent with certain antimetabolites that decrease the chance of cancer relapse or recurrence [90] (Table 10).

**Table 10:** Combinatorial therapeutic options for BC treatment

Polychemotherapy agents	Effects	Refs.
Neoadjuvant epirubicin + docetaxel	Feasible for patients with BC showing unfavourable conditions like locally advanced breast carcinoma and inflammatory breast carcinoma	[20]
Docetaxel + preoperative doxorubicin and cyclophosphamide	Better pathological and clinical response in cases of operable cancers	[91]
Gemcitabine + paclitaxel + epirubicin + cyclophosphamide Taxanes before anthracyclines	No improved pathological complete response (pCR) Improves pCR in neoadjuvant chemotherapies	[92]
Cyclophosphamide + methotrexate + 5-fluorouracil (5-FU), also called the ‘CMF regimen’	Showed increased disease-free survival and overall survival	[90]
Paclitaxel + cisplatin	Better treatment results for metastatic BC	[93]
Docetaxel + doxorubicin	Effective against locally advanced and metastatic BC	[94]
Docetaxel + epirubicin	Treats locally advanced BC due to reduced tumour RN integrity	[95]
Docetaxel + cisplatin	Effective in patients with anthracycline-resistant metastatic BC	[96]
Low-dose mitomycin + low-dose weekly doxorubicin with prior treatment with CMF	Better treatment of metastatic BC	[97, 98]
Gemcitabine + capecitabine + vinorelbine + taxanes	Treatment of aggressively progressing HER2 + metastatic BC	[99]
Panitumumab + gemcitabine + carboplatin	Better treatment of metastatic TNBC	[100]
Heat shock protein 90 (Hsp90) inhibitors + 17-allylamino + 17-demethoxygel-danamycin (17-AAG) + anticancer drugs like taxanes, cisplatin, etoposide and trastuzumab tanespi-mycin (17-AAG) + trastuzumab	HER2 + metastatic BC treatment Antitumor activity in HER BC where tumour progressed after trastuzumab treatment	[101] [102]
Pertuzumab + trastuzumab + docetaxel	Improved overall survival in metastatic BC patients	[103, 104]

### Significance of the findings

The results presented in the manuscript gives a comprehensive account of non-coding RNAs (ncRNAs), i.e., microRNAs, long non-coding RNAs (lncRNAs), and circular RNAs (circRNAs), in the management of metastatic breast cancer (MBC). These highlight the regulatory functions exerted by ncRNAs on a range of processes that are at the center of metastasis, such as epithelial-mesenchymal transition, angiogenesis, immune evasion, and drug resistance. Nevertheless, in spite of the fact that the manuscript compiles an unimaginable volume of mechanistic and experimental evidence defining the therapeutic potential of ncRNAs, there are certain limitations and shortcomings in the existing body of evidence.

### Limitations of the review

A significant limitation is the overwhelming reliance on in vitro and animal model research, with an equally remarkable lack of clinical trials or translational studies validating the safety and efficacy of ncRNA-targeted therapies in human patients. This shortcoming prevents the timely application of the findings in clinical practice. In addition, a significant number of the cited studies are based on limited sample sets, are geographically or demographically limited, and at times lack comprehensive longitudinal validation, and thus are subject to sampling and publication biases.

A further crucial ignorance concerns the limited appreciation of the ncRNA interaction within complex networks of molecules. Although individual ncRNAs can be tied to individual signaling pathways, the integrated regulatory environment—involving feedback, mutually competing endogenous RNA (ceRNA) networks, and epigenetic modification—is commonly schematized far too reductionistically. Moreover, context-dependent variability across breast cancer subtypes and subtype heterogeneity is not always considered, with potential for misleading interpretation and generalizations toward therapeutic targets.

### Future directions

The potential of these findings to inform future research is substantial. They highlight the importance of integrative multi-omics strategies and more advanced models that simulate human metastatic microenvironments. Clinically, if confirmed by rigorous trials, ncRNAs may be used as minimally invasive biomarkers for the early detection of metastasis or as new drug targets to bypass resistance mechanisms. Personalized medicine strategies that include ncRNA profiling can potentially transform patient stratification and treatment planning. However, the translation of these findings into clinical applications will necessitate interdisciplinary collaborations, standardized protocols, and rigorous regulatory evaluations.

## Conclusion

Even though tremendous progress has been achieved in the diagnosis and treatment of metastatic BC, drug resistance remains a formidable barrier. The present review has put the spotlight on the critical role of non-coding RNAs in facilitating resistance; however, it is necessary to appreciate that several intersecting biological processes contribute to therapeutic failure. Mechanisms such as efflux transporter overexpression, target gene mutation, increased DNA repair pathways, tumor microenvironment modulation, epithelial-to-mesenchymal transition, and metabolic reprogramming all contribute to drug resistance across various BC subtypes. In the future, studies must embrace an integrative systems biology approach to unravel the intricate processes that occur between these resistance mechanisms. This must include the application of high-throughput sequencing, CRISPR-based functional screens, and multi-omics analyses to identify novel drivers of resistance and actionable biomarkers. Moreover, the creation of organoid models and PDXs can also facilitate the laboratory-clinical gap, thereby enabling precise predictions of drug responses. Clinically, precision oncology methodologies that personalize treatments according to individual patient profiles—genomic and transcriptomic information—become increasingly relevant. The intersection of next-generation drug delivery systems, e.g., nanocarriers and exosome-mediated transport, with rational drug combinations is also poised to overcome resistance and enhance outcomes. Ultimately, the elimination of metastatic BC would call for a coordinated effort that unites molecular biologists, oncologists, pharmacologists, and data scientists. As the understanding of resistance mechanisms expands, the integration of personalized medicine principles with leading-edge therapeutic technologies has the potential to translate into more sustained responses and improved survival rates for patients suffering from metastatic BC.

## Data Availability

No datasets were generated or analysed during the current study.
